# Therapeutic Potential of Neurotrophins for Repair After Brain Injury: A Helping Hand From Biomaterials

**DOI:** 10.3389/fnins.2019.00790

**Published:** 2019-08-02

**Authors:** Josh Houlton, Nashat Abumaria, Simon F. R. Hinkley, Andrew N. Clarkson

**Affiliations:** ^1^Brain Health Research Centre, Department of Anatomy, University of Otago, Dunedin, New Zealand; ^2^State Key Laboratory of Medical Neurobiology, MOE Frontiers Center for Brain Science, Institute of Brain Science, Fudan University, Shanghai, China; ^3^Department of Laboratory Animal Science, Shanghai Medical College, Fudan University, Shanghai, China; ^4^The Ferrier Research Institute, Victoria University of Wellington, Petone, New Zealand

**Keywords:** neurotrophins, BDNF, Trk receptor, p75 neurotrophic receptor, hydrogel, microsphere encapsulation, electrospun fibers, HSPG

## Abstract

Stroke remains the leading cause of long-term disability with limited options available to aid in recovery. Significant effort has been made to try and minimize neuronal damage following stroke with use of neuroprotective agents, however, these treatments have yet to show clinical efficacy. Regenerative interventions have since become of huge interest as they provide the potential to restore damaged neural tissue without being limited by a narrow therapeutic window. Neurotrophins, such as brain-derived neurotrophic factor (BDNF), and their high affinity receptors are actively produced throughout the brain and are involved in regulating neuronal activity and normal day-to-day function. Furthermore, neurotrophins are known to play a significant role in both protection and recovery of function following neurodegenerative diseases such as stroke and traumatic brain injury (TBI). Unfortunately, exogenous administration of these neurotrophins is limited by a lack of blood-brain-barrier (BBB) permeability, poor half-life, and rapid degradation. Therefore, we have focused this review on approaches that provide a direct and sustained neurotrophic support using pharmacological therapies and mimetics, physical activity, and potential drug delivery systems, including discussion around advantages and limitations for use of each of these systems. Finally, we discuss future directions of biomaterial drug-delivery systems, including the incorporation of heparan sulfate (HS) in conjunction with neurotrophin-based interventions.

## Introduction

Stroke consistently remains a leading cause of death and disability worldwide (Mozaffarian et al., 2015). While stroke preferentially affects older people, stroke can affect anyone of any age, race, or gender (Feigin et al., 2014). In addition, ethnic disparities are known to exist, for instance, New Zealand Māori, and Pacific Island populations experience a stroke at a much younger age than Europeans (Feigin et al., 2006). With the global population continuing to age, the number of people suffering from a stroke and subsequently living with a lasting disability is also expected to rise. As a result, there is an urgent and unmet need to find treatment options to aid in improving recovery of lost functions.

The central nervous system CNS has a limited capacity to regenerate, which is one of the main causes for why stroke patients recover poorly. In addition, there is a lack of spontaneous recovery seen after stroke, leading to a large personal, and societal burden (Mozaffarian et al., 2015). This burden has led research into investigating neural mechanisms of regeneration and repair, in the hope to restore and improve lost functions following stroke. Depending on the neuronal area affected by the stroke (i.e., motor, speech, or language centers), rehabilitation, mental practice, and music therapies have all been shown to increase the capacity to recover after a stroke (Johansson, 2011). However, in patients who have had a large stroke, rehabilitation is less effective in facilitating an improvement in functional recovery (Johansson, 2011).

Significant work has shown that exogenous administration of GFs can help facilitate the repair of injured CNS tissue via their ability to regulate neuronal growth and survival (Connor and Dragunow, 1998; Sofroniew et al., 2001; Berretta et al., 2014). In particular, BDNF has been highlighted as being a key regulator of rehabilitation-induced recovery after stroke (Ploughman et al., 2007, 2009). Moreover, activity-driven increases in BDNF have also been shown to promote motor recovery after stroke (Fritsch et al., 2010; Clarkson et al., 2011).

The regenerative capabilities of neurotrophin-mediated interventions have already been observed in preclinical models of neurodegenerative diseases. Specifically, systemic administration of BDNF, NGF, and NT-3 have been reported to enhance neurite outgrowth, neurogenesis, and functional recovery in rodent models of stroke (Grill et al., 1997; Jakeman et al., 1998; Ramer et al., 2000; Winkler et al., 2000; Schäbitz et al., 2004, 2007). However, the translation of such treatments into a clinical setting has been challenged by poor BBB permeability, off-target effects on the PNS, and short half-life (Chan et al., 2017). Approximately 98 percent of all compounds targeting the CNS have failed to cross the BBB (Pardridge, 2005), creating a need for alternative approaches. As a novel solution to this issue, various biomaterials have been engineered to provide an effective, and sustained drug-delivery system to the injured brain. These systems allow stem cells and/or small drug molecules including neurotrophins to bypass the BBB (Aizawa et al., 2008) and be delivered directly to the site of injury.

Heparan sulfate, as the GAG cleaved from its protein backbone, is thought to provide a novel approach for the delivery of neurotrophins to the injured CNS. HS plays a critical role in coordinating GFs and mediating their biological potential (Xu and Esko, 2014). This highly sulfated biopolymer is ideally suited as a component of a biomaterial therapeutic for brain injury. It is chemically stable, physiologically tolerated, has a demonstrated long-term benefit to wound repair by tunable protein-interactions and has been demonstrated to tolerate the level of γ-irradiation treatment required to sterilize sufficiently medical-device-like materials (Smith et al., 2018). In addition, HS has demonstrated a remarkable propensity to stabilize short-lived GF’s in what appears to be a selective fashion (Wang et al., 2014; Wijesinghe et al., 2017).

The effects of BDNF and other GFs have previously been investigated in various stroke models and have shown strong regenerative potential (Berretta et al., 2014). In general, BDNF is believed to have a beneficial effect on stroke recovery via several mechanisms: protection against acute ischemic injury (Schäbitz et al., 2007), increased angiogenesis (Kermani and Hempstead, 2007), neurogenesis (Schäbitz et al., 2007), and neural repair (Mamounas et al., 2000) as well as enhanced synaptic plasticity (Waterhouse and Xu, 2009; Clarkson et al., 2011). Whilst the use of BDNF and other GFs to promote neuroprotection and minimize the spread of damage during the acute phase of injury has been extensively studied (Pardridge, 2005; Wu, 2005; Cai et al., 2014), this work will not be reviewed here. This review instead summarizes past experimental evidence that highlights how neurotrophins have shown potential as a delayed treatment option for aiding in repair and regeneration of neural tissue for stroke and other neurodegenerative diseases. In addition, we discuss novel biomaterial delivery systems that have been utilized to enhance the delivery of neurotrophins to the CNS, as utilizing such delivery systems have resulted in a further improvement in functional recovery when treatment is delayed by days to weeks post injury.

### Stroke Pathophysiology and Mechanisms of Endogenous Repair

Stroke occurs when blood flow to the brain is either obstructed by an occlusion (ischemic) or following rupturing (hemorrhagic) of a cerebral blood vessel. Lack of oxygen and glucose, and a build-up of toxic by-products, cause the areas of the brain deprived of blood flow to undergo a chain of pathological events (ischemic cascade), ultimately leading to cell death, and loss of function associated with that region of the brain (Dirnagl et al., 1999). As time passes, a lack of reperfusion to the penumbra (regions surrounding the core infarct) results in further expansion until the stroke is fully formed. Whilst neuroprotective agents have shown preclinical success at restoring reperfusion and minimizing cellular damage, they are only effective when administered within the first few hours following stroke onset and have failed to translate into clinical use, with the exception of thrombolytic compounds (Moretti et al., 2015). As a result, much needed stroke research unraveling the mechanisms associated with neuroregeneration and repair in the days to weeks following a stroke have highlighted a number of targetable treatment options, some of which have already resulted in the establishment of clinical trials.

Whilst the CNS shows limited capacity for regeneration and repair, under pathological conditions such as stroke, axonal sprouting, endogenous neurogenesis, and spontaneous functional recovery appear to be enhanced (Carmichael et al., 2001; Benowitz and Carmichael, 2010). In addition, Stroemer et al. (1995) reported that both GAP-43 and synaptophysin, two proteins involved with neurite growth and synaptogenesis; are upregulated throughout the neocortex 2 weeks following a focal infarct in rats. Further, these researchers show a positive correlation between improved locomotor function and stroke-induced elevations in GAP-43 and synaptophysin. It has since been established that enhancing neuronal connections throughout the ischemic brain via pharmacological interventions correlates with an improvement in functional motor recovery after stroke (Lee et al., 2004; Overman et al., 2012; Clarkson et al., 2013; Cook et al., 2017).

Neurogenesis occurs continuously in two areas of the healthy adult CNS: the SVZ of the lateral ventricle, and the subgranular zone (SGZ) of the hippocampal dentate gyrus. In these areas, self-renewing multipotent cells known as NSPCs differentiate into both neuronal, and glial cells (Taupin, 2006). Under physiological conditions these NSPCs migrate to the dentate gyrus or olfactory bulb to replenish the continuously dying granule cells or olfactory neurons, respectively. Following acute injury to the CNS, these resident neural progenitors may also be induced in an attempt to replace damaged neurons (Zhang et al., 2005; Greenberg, 2007). Supporting this notion, many animal models of stroke have reported the upregulation of SVZ and SGZ neurogenesis, peaking around 7–10 days post-stroke (Iwai et al., 2002, 2003; Parent et al., 2002; Carmichael et al., 2005). Furthermore, stroke-induced local changes to the microvasculature has been found to attract neuroblasts generated within the SVZ and facilitate their migration to the ischemic boundary zone (peri-infarct), an area lying lateral to the stroke (Carmichael et al., 2005; Yamashita et al., 2006; Thored et al., 2007).

Interestingly, preclinical research indicates NSPCs offer the majority of their regenerative potential through bystander effects, specifically by providing a rich source of GFs to the injured tissue rather than contributing to structural reconstruction (Chen et al., 2000). Supporting this, preclinical work has shown that MSCs facilitate an improvement in functional recovery, even though they fail to differentiate into mature neurons or glia when grafted into the ischemic boundary zone of rats exposed to a MCAo (Chen et al., 2000). Moreover, at least a portion of the therapeutic potential of MSCs has been ascribed to their ability to enhance endogenous neurogenesis and protect newborn cells from deleterious environments (Yoo et al., 2008), both of which are mechanisms mediated by neurotrophin signaling pathways. Although explicit evidence directly linking enhanced neurogenesis and functional recovery is sparse, a direct causal relationship seems probable with almost all neuroregenerative agents that improve neurological function following stroke also potentiating neurogenesis (Zhang et al., 2001; Wang et al., 2004; Schäbitz et al., 2007; Cook et al., 2017). Of note, HS has also been demonstrated to support the proliferation (without differentiation) of stem cells *ex vivo* (Dombrowski et al., 2009; Wijesinghe et al., 2017).

In addition to changes in neurogenesis, angiogenesis and axonal sprouting, work over the past few years has highlighted a chronic imbalance in brain excitability following stroke (Clarkson, 2012). Specifically, stroke has been reported to weaken transcallosal inhibition from the ipsilesional hemisphere onto the intact, contralesional hemisphere (Murase et al., 2004; Duque et al., 2005). This further exacerbates an increase in tonic GABAergic inhibition onto the stroked hemisphere, limiting its potential for cortical plasticity, and spontaneous recovery after stroke (Clarkson et al., 2010). Indeed, restoring neuronal excitability in the ipsilesional stroke hemisphere via increasing AMPA activity, and/or dampening tonic GABAergic inhibition has shown great promise to enhance recovery of lost function (Clarkson et al., 2010, 2015; Orfila et al., 2017). Whilst the above evidence collectively supports an intrinsic capability of the ischemic brain to regenerate and repair itself, it must be reiterated that these mechanisms alone are most often insufficient to cause a complete reversal of the functional impairment (Yamashita and Abe, 2012). Furthermore, age-related decreases in neurogenesis (Cooper-Kuhn et al., 2004; Kernie and Parent, 2010) and angiogenesis (Sonntag et al., 1997) have been reported, making it even harder for endogenous repair mechanisms to overcome damage and promote recovery. Accordingly, regenerative interventions have begun to target the potentiation of these endogenous mechanisms to try and maximize functional recovery in animal models of stroke and other neurodegenerative conditions such as SCI and Alzheimer’s disease (Wang et al., 2004; Piantino et al., 2006; Schäbitz et al., 2007; Weishaupt et al., 2014; Choi et al., 2018). This has been demonstrated in the case of the endogenous stimulation of key GF’s by addition of HS on a biomaterial support to the wound site (Murali et al., 2013).

### Neurotrophins for Repair and Regeneration

**FIGURE 1:**
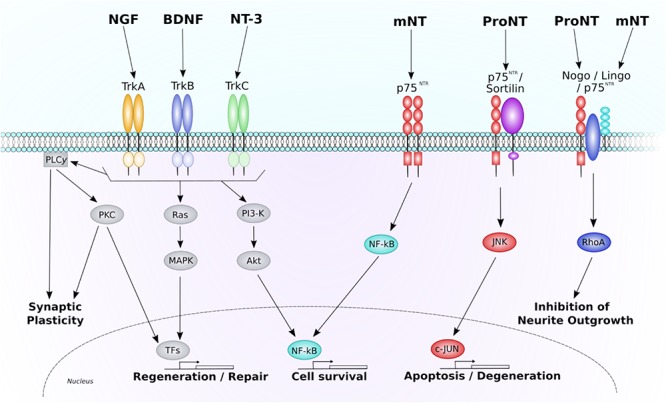
Neurotrophin signaling through the p75^NTR^ and Trk receptors. This diagram depicts the major intracellular signaling pathways associated through each neurotrophin receptor. Each Trk receptor isoform binds mature neurotrophins and acts through three predominant pathways. Activation of PLC-γ1 results in PKC-mediated promotion of synaptic plasticity. Activation of Ras initiates MAPK-mediated promotion of neuronal regeneration and growth. Activation of PI3-K results in activation of Akt and promotion of NF-κB-mediated cell survival. Each of these pathways are also known to regulate genetic transcription, further promoting pro-survival, and regenerative gene expression. The p75^NTR^ receptor also regulates three main pathways. When a mature neurotrophin binds to an isolated p75^NTR^ NF-κB-mediated cell survival is promoted. If the p75^NTR^ is co-expressed with the sortilin receptor, pro-neurotrophins can bind, and cause activation of JNK-c-Jun mediated cell death and degeneration. A receptor complex consisting of Nogo, p75^NTR^, and Lingo1 can bind both pro- and mature-neurotrophins to alter neurite outgrowth in a RhoA-dependent manner. NGF, nerve growth factor; BDNF, brain-derived neurotrophic factor; NT-3, neurotrophin-3; Trk, tropomyosin receptor kinase; mNT, mature neurotrophin; ProNT, proneurotrophin; p75^NTR^, pan neurotrophin receptor 75; PLC*y1*, phospholipase C gamma one; PKC, protein kinase C; MAPK, mitogen-activated protein kinase; TFs, transcription factors; PI3-K, phosphoinositide 3-kinase; NF-kB, nuclear factor kappa-light-chain-enhancer of activated B cells; JNK, c-Jun N-terminal kinase.

Neurotrophins are the predominant mediators of neuronal survival and regeneration throughout the CNS, making them of particular interest in neuroregenerative research. The neurotrophin family consists of several main GFs, including NGF, BDNF, and NT-3. All neurotrophins are initially synthesized as precursor proteins, known as pro-neurotrophins. These pro-neurotrophins can then be cleaved intracellularly by furin or proconvertases, or extracellularly by metalloproteases and plasmin, to form stable mature neurotrophins. Whilst mature neurotrophins selectively bind to their respective Trk to exert neurotrophic effects, pro-neurotrophins have been found to conduct somewhat opposing, pro-apoptotic effects through the p75^NTR^/sortilin receptor (see Figure 1: further information on neurotrophin signaling pathways can be found below; (Nykjaer et al., 2004; Teng et al., 2005). This finding has left the scientific world moving away from the passive function of these pro-domains, and has brought a new level of complexity to the role and function of neurotrophin signaling in the CNS (Zanin et al., 2017).

The expression of both neurotrophins and their respective receptors typically decrease with age, indicating neurons and glia yield a limited trophic ability to combat natural, and pathological neurodegeneration (Webster et al., 2002; Lommatzsch et al., 2005). In support of this, reduced BDNF expression in the hippocampus has been shown to correlate with impairments in both spatial and working memory in gerbils and rats (Schaaf et al., 2001; Hwang et al., 2006). Furthermore, excitotoxic lesions to the nigrostriatal pathway increases BDNF expression in the striatum of young, but not aged rats (Yurek and Fletcher-Turner, 2000, 2001). Similarly, lesions to the medial septum significantly elevates hippocampal levels of NGF in young rats only (Scott et al., 1994). Interestingly, further assessment of these young rats revealed an enhancement in neurite outgrowth compared to their aged counterparts (Scott et al., 1994; Kaseloo et al., 1996). Evidence from preclinical animal models also supports an age-related difference in expression of BDNF, with stroke inducing a lessened elevation of BDNF in aged mice compared to young stroke controls (Clarkson et al., 2015). In summary, aging exacerbates the degree of functional impairment as a consequence of damage to the brain, relative to young animals. This is thought to be in part, due to the differing recovery trajectories observed in aged and young animals, including differences in spontaneous recovery, neurogenic activity, and the neuronal sprouting transcriptome (Li S. et al., 2010; Hermann and Chopp, 2012). Therefore, it has been postulated that age-specific interventions may be required to maximize the effectiveness of regenerative interventions. Specifically, aged animals may require a combinatorial intervention to gain functional recovery to the same extent as young mice. Supporting this, systemic administration of the high impact AMPAKine (positive allosteric modulator of AMPA receptors), CX1837, has been reported to stimulate an increase in BDNF expression and promote functional recovery in aged mice following photothrombotic strokes (Clarkson et al., 2015). Interestingly, the use of low impact AMPAkines (positive allosteric modulator of AMPA receptors) have failed to elicit functional recovery in the same animal model (Clarkson et al., 2011). Although neurotrophins mediate intrinsic repair mechanisms, the above collectively supports that their intrinsic regeneration abilities decline during aging.

### Neurotrophin Signaling Pathways

The neurotrophin family shares two common classes of transmembrane receptors; the Trk receptors that bind the mature neurotrophins, and the p75^NTR^, a receptor from the tumor necrosis factor (TNF) receptor superfamily that preferentially binds the pro-neurotrophins. Whilst the low-affinity p75^NTR^ binds to all members of the neurotrophin family with similar nanomolar affinities (Bothwell, 1995), each neurotrophin shows preference for different Trk receptor isoforms; specifically, NGF binds to the TrkA, BDNF to TrkB, and NT-3 to TrkC. Once a mature neurotrophin has bound to its respective Trk receptor, the Trk receptor dimerizes and undergoes transphosphorylation to specific intracellular tyrosine kinase residues. These phosphorylated tyrosine-kinase residues act as docking sites for adaptor proteins that allow additional kinases to be recruited for activation of intracellular signaling pathways (see Figure 1).

Activation of the Trk receptor is known to phosphorylate and activate PLC-γ1 and its associated second messenger signaling pathway. PLCγ activity generally generates two distinct second messenger systems: IP3 and DAG, both of which increase intracellular release of calcium (Ca^2+^) and stimulate PKC-mediated signaling, resulting in enhanced neuronal and synaptic plasticity (Chao, 2003; Yoshii and Constantine-Paton, 2010). Trk receptors have also been found to activate Ras, a small GTP-binding protein that further activates the MAPK signaling pathway to generate ERK. ERK then phosphorylates and activates a range of TFs including CREB, causing downstream transcriptional changes to genes that control neuronal differentiation and neurite outgrowth (Kaplan and Miller, 2000). Another major pathway activated by Trk receptors is the PI3-K pathway, which leads to downstream activation of the serine/theronine kinase, AKT to promote NF-kB-mediated neuronal survival (Reichardt, 2006). The PI3K/AKT signaling pathway plays a central role in regulating cell growth, proliferation, and survival under physiological and pathological conditions (Cantley, 2002). In addition, PI3K/AKT signaling has been revealed to contribute to axonal sprouting in cultured hippocampal neurons (Atwal et al., 2000; Sanchez et al., 2001; Jaworski et al., 2005), which is an important mechanism underlying post-stroke functional recovery (Li S. et al., 2010; Overman et al., 2012; Clarkson et al., 2013). For instance, in a recent paper by Clarkson et al. (2015), experimenters combined systemic administration of a high impact AMPAKine, CX1837, and local hydrogel delivery of BDNF, resulting in a synergistic increase in phosphorylation of AKT, MEK, and CREB in aged (22–24 months old) mice. This upregulation was observed in parallel to increased functional recovery of motor function. In addition, recent work has also shown that modulation of GSK-3 after stroke can enhance axonal outgrowth (Ueno et al., 2012). Activation of the AKT/GSK-3/CREB pathway has also been observed in other age-related neurological disorders such as Alzheimer’s disease, with activation of this pathway leading to an improvement in cognitive function (Tang et al., 2014). To this end the full complement of Trk-mediated signaling pathways that play a role in post-stroke recovery, or their involvement in mechanisms associated with recovery, still remain to be full elucidated.

Whilst neurotrophins signal via two different receptor pathways, most of the attention has been focused on the activation of Trk receptors and their subsequent biological effects (see Figure 1). However, over the past couple of decades research has also begun to investigate the surprisingly diverse functions of p75^NTR^, including a somewhat paradoxical involvement in pro-apoptotic signaling (Reichardt, 2006). Most neuronal populations that respond to neurotrophins co-express both p75^NTR^ and Trk receptors. Whilst the exact mechanism remains elusive, the interaction between these two receptors is known to positively modulate Trk function following binding of a mature neurotrophin (Reichardt, 2006). Contrastingly, in the absence of Trk, p75^NTR^ can also promote apoptotic signaling when a complex is formed with sortilin, a Vps10-domain containing protein that acts as a co-receptor for pro-neurotrophin binding (Nykjaer et al., 2004; Teng et al., 2005). In this scenario the activated pro-neurotrophin-receptor complex activates Rac, a GTP-binding protein that promotes the JNK cascade (Harrington et al., 2002), leading to activation of pro-apoptotic genes and apoptotic cell death (Reichardt, 2006).

The p75^NTR^ itself lacks any catalytic intracellular domains, however, it is able to signal via several adaptor proteins. Specifically, p75^NTR^-mediated recruitment of NRIF1/NRIF2, NADE and SC-1 can activate several apoptotic signals including Rac, JNK and NF-kB (Mukai et al., 2000; Dechant and Barde, 2002; Yeiser et al., 2004). The p75^NTR^ is also known to form a signaling complex with the transmembrane proteins Lingo-1 and Nogo-66 (NgR1) receptor, a receptor previously reported to inhibit axonal sprouting and neural plasticity (Schwab, 2010). Although it is not fully understood how, this receptor complex is known to activate the small GTPase protein, RhoA, leading to inhibition of oligodendrocyte differentiation and myelination, and inhibition of axonal growth in the CNS (Tan et al., 2011). Both pro-neurotrophins and mature neurotrophins have been reported to bind to the p75^NTR^/NgR1/Lingo1 receptor complex, with opposing effects on neurite growth being observed (Lehmann et al., 1999; Deinhardt et al., 2011; Sun et al., 2012). Specifically, introduction of proBDNF or proNGF to neuronal cultures induces growth cone collapse (i.e., the developing or regenerating tip of a neurite) and inhibition of further neurite extension (Lehmann et al., 1999; Deinhardt et al., 2011; Sun et al., 2012). Contrastingly, binding of mature forms of BDNF and NGF to this receptor complex appears to inhibit RhoA activity leading to subsequent disinhibition of neurite outgrowth (Yamashita et al., 1999). Collectively, these findings indicate bidirectional effects of p75^NTR^ depending on both the ligands and receptors present in a particular neuronal microenvironment. It must be reiterated, however, that the mature neurotrophins’ enhanced affinity for the Trk receptor counteracts the proapoptotic action of p75^NTR^ activity, resulting in the predominant function of neurotrophin being trophic.

### Nerve Growth Factor (NGF)

In the early 1950’s Levi-Montalcini (1987) first discovered NGF, demonstrating its’ ability to regulate the survival and maturation of developing PNS neurons. NGF has since become one of the best characterized members of the neurotrophin family, acting on many neuronal and glial cell populations throughout the nervous system to promote axonal sprouting, dendritic arborization, and cell body growth (Cuello, 1996; Debeir et al., 1999; Sofroniew et al., 2001). Moreover, recent work has highlighted an interaction between NGF and cells from the immune-hematopoietic linage, including mast cells (Skaper, 2017). Specifically, mast cells have been found to synthesize and release NGF, with NGF also acting back on mast cells to promote survival (Skaper, 2017). Further, NGF concentrations are elevated in various inflammatory and autoimmune states in humans, such as multiple sclerosis and chronic arthritis (Aloe et al., 1992; Laudiero et al., 1992). Whilst this area of research is in its infancy, these findings suggest that under certain conditions, NGF may be able to promote inflammatory cascades, and have deleterious effects on surrounding tissues, arguing a more complicated role of this neurotrophin than previously thought.

Various CNS injuries such as ischemia, seizure-activity and trauma induce a rapid upregulation in the expression of NGF and the TrkA receptor (Lindvall et al., 1994; Goss et al., 1998; Gottlieb and Matute, 1999; Dmitrieva et al., 2016). Although the exact function of this injury-induced upregulation is not fully understood, several lines of evidence suggest enhanced NGF signaling may facilitate the repair of damaged nervous tissue. For example, implantation of fibroblasts, genetically modified to secrete NGF reduce cholinergic cell death and promote axonal sprouting in the aged rats and non-human primates undergoing lesion-induced degeneration (Kawaja and Gage, 1991; Kordower et al., 1994). Moreover, exogenous NGF has also been reported to have a similar effect on transected SC axons, promoting regeneration of damaged axons and restoration of stimulus-driven electrophysiological activity (Ramer et al., 2000). However, it must be noted that the ability of NGF to promote functional recovery in animal models of neurodegenerative diseases remains largely understudied with somewhat opposing findings. For example, enhanced NGF-induced sprouting following SCI is known to be a primary cause of autonomic dysreflexia, a condition in which potentially life threatening episodic hypertension is triggered by stimulation of sensory nerves in the body below the site of injury (Krenz et al., 1999; Weaver et al., 2006). In these situations, enhanced NGF signaling stimulates sprouting of small-diameter primary afferent fibers, thereby amplifying SC reflexes, and promoting dysreflexia. For a more detailed discussion of NGF treatment’s mixed success for treating SCI see a recent review by Keefe et al. (2017).

The systemic administration of NGF in clinical trials has also revealed a variety of adverse side effects including marked reductions in body weight, and myalgia to the muscles of the trunk and back (Petty et al., 1994; Eriksdotter Jönhagen et al., 1998). Despite this, the topical administration of recombinant human NGF to the eyes of patients with ophthalmological diseases such as neurotrophic keratitis and dry eye disease has shown promise in relieving symptoms and promoting healing of the damage eye (Bonini et al., 2018; Rocco et al., 2018; Sacchetti et al., 2019).

### Brain-Derived Neurotrophic Factor (BDNF)

Brain-derived neurotrophic factor is the most abundant neurotrophin in the CNS (Andero et al., 2014). BDNF-TrkB signaling is well documented to regulate a wide range of neuronal functions including cell survival, neuronal differentiation and migration, neurite outgrowth, and facilitation of LTP and plasticity (Encinas et al., 1999; Boyd and Gordon, 2003b; Pang and Lu, 2004). Both BDNF and its high affinity receptor, TrkB, are widely produced throughout the CNS with high expression being observed in areas such as the neocortex, hypothalamus, and amygdala (Connor and Dragunow, 1998; Binder and Scharfman, 2004).

Studies using *in situ* hybridization have revealed a stroke-induced upregulation of BDNF mRNA expression in both the ipsilateral cortex and hippocampus in rat models of forebrain ischemia (Comelli et al., 1993; Kokaia et al., 1995; Dmitrieva et al., 2016). This upregulation appears to peak within the first 12 h after stroke and remains elevated as late as 1 week later (Béjot et al., 2011; Clarkson et al., 2011). Indeed, stroke-induced changes in BDNF protein have also been observed, with microsphere embolism-induced MCAo causing elevated BDNF expression over a similar time course (Miyake et al., 2002). However, these changes appear to vary across brain regions, with a suppression in BDNF protein expression being observed in the parietal cortex 24-h after MCAo in rats (Kokaia et al., 1995). The expression of BDNF and TrkB levels are also modulated in animal models of SCI (Ikeda et al., 2001) and traumatic brain injury (TBI), which occurs following blunt or penetrating forces to the skull causing damage to white matter tracts, intracranial hemorrhaging, and ischemia (Rostami et al., 2014).

It must be noted, however, that stroke-induced increases in endogenous BDNF are insufficient to reverse functional impairments (Li B. et al., 2010). Supplementing the ischemic brain with exogenous BDNF has thus been investigated over the past few decades, with research supporting BDNF-enhanced axonal sprouting (Mansour-Robaey et al., 1994; Batchelor et al., 2000, 2002; Mamounas et al., 2000), angiogenesis (Kermani and Hempstead, 2007), neurogenesis (Schäbitz et al., 2007), neuronal plasticity (Yacoubian and Lo, 2000; Zakharenko et al., 2003), and behavioral recovery (Schäbitz et al., 2004; Guan et al., 2012). Importantly, several studies have also reported that a critical threshold in BDNF activity is required in order to achieve an improvement in functional recovery in the weeks following stroke (Ploughman et al., 2009; Clarkson et al., 2011; MacLellan et al., 2011). This was first shown following enriched rehabilitation has been found to only improve motor recovery in rats with elevated BDNF levels post-MCAo (MacLellan et al., 2011). Furthermore, suppressing endogenous BDNF translation with antisense oligonucleotides prevents such improvements (Ploughman et al., 2009). Clarkson et al. (2011) further demonstrated functional motor recovery in mice following administration of a BDNF-inducing AMPAkine following photothrombotic stroke. This effect was not observed when a non-BDNF inducing AMPAkine was applied, or when BDNF function was inhibited with the BDNF ligand decoy, TrkB-Fc (Clarkson et al., 2011). Interestingly, BDNF and the TrkB receptor is abundantly expressed throughout the sensory neurons in the inner ear, leading many researchers to investigate the potential of BDNF in treating deafness (Ramekers et al., 2012). For example, Shibata et al. (2010) treated neomycin-deafened rats with transgenic vectors overexpressing the gene for BDNF within the BMA of the inner ear. Results showed that treatment with the BDNF vectors could promote regrowth of damaged nerve fibers into the cochlear epithelium and enhance survival of spinal ganglion neurons.

Given neurotrophic effects of BDNF it is reasonable to presume that the injury-induced upregulation of BDNF and is receptor acts to restrict pro-apoptotic signaling following injury. Further support for this notion lies in clinical studies investigating a common SNP of the BDNF gene, where valine is substituted for methionine at codon 66 (BDNF val*66*met, otherwise known as rs6265). A resulting 19–30% reduction in activity-dependent secretion of BDNF protein has been reported in humans carrying this SNP (Shimizu et al., 2004), with worse functional outcomes being reported in these individuals following a stroke (Siironen et al., 2007; Mirowska-Guzel et al., 2012). Be that as it may, the val*66*met allele has also been reported to promote recovery of executive function in individuals with TBI (Krueger et al., 2011). Moreover, knock-in-mice homozygous for the Met allele have been reported to display enhanced functional recovery following chronic stroke (Qin et al., 2014). These findings argue a more complicated role of this SNP that requires future investigation to clarify its role following CNS injury.

To date, only a few studies have conducted clinical trials investigating BDNF as a therapeutic intervention. Both intrathecal and subcutaneous administrations of BDNF have been found to be well tolerated in ALS patients (Beck et al., 1994; Bradley, 1999; Ochs et al., 2000). The neurorehabilitative potential of BDNF, however, was not observed due small cohort size and limited half-life. Other proposed factors limiting BDNF’s success in human trials includes the difficulty of producing sufficient amounts of BDNF for use in human interventions, and the lack of studies investigating higher doses of BDNF in animal models (Nagahara and Tuszynski, 2011). However, the use of biomaterial drug-delivery systems holds promise in circumventing these issues, as lower concentrations of BDNF can be administered directly to the brain without worrying about BBB passage as discussed below.

### Neurotrophin-3 (NT-3)

Neurotrophin-3 is another member of the neurotrophic family, sharing 55 percent amino acid homology with BDNF, and NGF (Maness et al., 1994). Contrasting to other members of the neurotrophin family, NT-3 expression within the CNS appears to peak at the time of fetal development, during which time NT-3 plays a strong role in neuronal survival, and differentiation (Ernfors et al., 1990; Maisonpierre et al., 1990; Ghosh and Greenberg, 1995). NT-3 conducts most of its trophic effects through a high affinity interaction with the TrkC receptor (Lamballe et al., 1991). However, at high concentrations NT-3 is also known to interact with other Trk isoforms at a much lower affinity compared to that of NGF and BDNF (Clary and Reichardt, 1994; Strohmaier et al., 1996).

Maisonpierre et al. (1990) first reported in 1990 that induction of the NT-3 gene facilitated neurite outgrowth in DRG explants. NT-3 has since been reported to mediate aspects of neurogenesis, with NT-3 neutralizing antibodies, and conditional NT-3 knockouts both causing significant reductions in NSPC differentiation and survival (Ghosh and Greenberg, 1995; Barnabé-Heider and Miller, 2003; Shimazu et al., 2006).

Contrasting with the other members of the neurotrophin family, NT-3 mRNA expression is significantly reduced in the first 12–24 h following injury to the CNS (Kokaia et al., 1995; Boris-Möller et al., 1998; Boyd and Gordon, 2003a). This is likely an intrinsic mechanism to help minimize damage, as conditional NT-3 knockout animals and animals administered NT-3 neutralizing antibodies have smaller infarct volumes following MCAo stroke and enhanced survival of axotomized corticospinal neurons, respectively, indicating a detrimental role of NT-3 in the acute phase of injury (Giehl and Tetzlaff, 1996; Bates et al., 2002). However, contrasting reports show intrathecal and intraventricular administration of NT-3 promote neurite extension, axonal sprouting, and neuronal survival when administered immediately after trauma to the SC (Schnell et al., 1994; Bradbury et al., 1999). Based on these findings, it is possible that NT-3 has differing rolls in both the CNS and PNS after injury, however, further work is required to confirm this hypothesis.

Contrary to findings showing early administration of NT-3 to the CNS being damaging, delayed administration of NT-3 (>3 days) after stroke appear to promote protection and repair (Zhang et al., 1999). NSCs engineered to overexpress NT-3 have been found to enhance neuronal differentiation in the penumbra and infarct cavity when administered to mice 3 days after hypoxic-ischemic injury (Park et al., 2006). Furthermore, Duricki et al. (2015, 2018) showed that intramuscular injections or prolonged subcutaneous infusion of human recombinant NT-3, given 24 h after endothelin-1 and MCAO-induced stroke, can enhance sensorimotor recovery in elderly rats. Interestingly, anterograde tracing revealed NT-3-enhanced neuroplasticity in the CST, with increased numbers of fibers extending from the intact to affected side. This change in plasticity has also been reported by Weishaupt et al. (2014) in a rat model of SCI, with enhanced axonal sprouting being observed from injured CST axons following intrathecal injections of a NT-3 expressing AAV.

To the best of our knowledge, the therapeutic potential of NT-3 has not been investigated in clinical trials for neurodegenerative CNS diseases (Bezdjian et al., 2016). However, phase I and II clinical trials in neurologically healthy adults have confirmed that NT-3 administration is safe and well tolerated (Chaudhry et al., 2000; Parkman et al., 2003). Furthermore, a pilot clinical study showed potential for the subcutaneous administration of NT-3 in patients with CMT1A, a PNS neuropathy resulting from a mutation to a gene for peripheral myelin protein in Schwann cells (Sahenk et al., 2005). Results revealed that patients treated with NT-3 showed significant improvement in the Mayo Clinic NIS and sensory modality function, compared to those treated with a placebo. It is hypothesized that these behavioral changes occur as a result of NT-3-mediated regeneration of sensory nerves. Whilst these findings do support the potential of NT-3 use clinically, larger cohort studies are required.

### Biomaterial-Based Delivery of Neurotrophins

It is already well established that neurotrophins play a major role in regulating neuroregenerative processes and facilitating an improvement in functional recovery in various animal models of neurodegenerative diseases. However, the translation of growth factor-based treatments into the clinic has been challenged by extremely poor BBB permeability, short therapeutic half-life and undesirable PNS side-effects, such as bone pains and increased hematocrit (Zhang et al., 2001; Ren and Finklestein, 2005; Lanfranconi et al., 2011; Chan et al., 2017). In 2006, an international panel of 44 experts conducted a forefront study identifying the top ten most promising applications of regenerative medicine for improving health in developing countries (Greenwood et al., 2006). Applications were evaluated for disease burden, therapeutic impact, feasibility, affordability, acceptability, and indirect benefits. The use of growth factor-seeded scaffolds for directing and enhancing SC and PNI was named in the top ten. Since then, it has become evident that this technology can be effectively applied into rehabilitative and regenerative approaches in other CNS injuries and diseases, in the hope to overcome the barriers faced by systemic administration.

The term biomaterial refers to a material that has been engineered to interact with a biological system, being tuned to hold various biodegradable, biocompatible, cytocompatible, physical, and topographical properties. The effect of altering these properties will not be included within the scope of this review, but the authors direct readers to the following review for their own reading (Führmann et al., 2017). Biomaterial scaffolds used in medical and scientific research are generally synthesized from biodegradable, aliphatic poly(esters) such as PLGA, PGA, PCL, PLA, PLLA, and poly(phosphoesters) (PPEs). These poly(esters) are preferentially used due to their excellent biocompatibility and biodegradability and their current FDA approval for use as a drug delivery system (Lewis, 1990). Proteinaceous scaffolds utilizing materials such as foamed-collagen (e.g., freeze-dried) also fulfill the requirements for biomedical implants that can exhibit controlled biodegradation.

**FIGURE 2:**
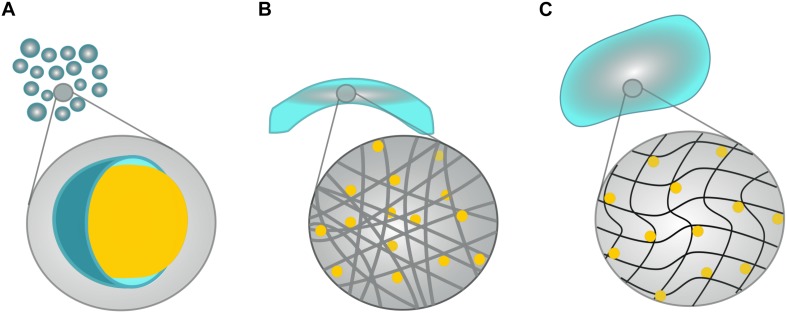
Schematic of possible biomaterial scaffolds. Although hundreds of biomaterials have been generated, they fall neatly into four main categories: **(A)** Microspheres are small, polymeric spheres (shown in blue) that encapsulate a target drug or molecule (as depicted by the yellow sphere). **(B)** Electrospun fibers consist of long, usually cylindrical, and fibers that are commonly crafted into tubes or conduits. Target drugs can either be bound to the fibers themselves, or left to freely diffuse from the fiber network. **(C)** Hydrogels consist of hydrophilic, polymeric networks that swell to conform to cavities formed by neurodegenerative diseases/injuries, and facilitate the passive release of target molecules.

When engineered specifically for stroke, biomaterials are typically designed to degrade in synchrony with the hosts healing process whilst supporting the efficacy of NSPCs grafts, and/or to amplify endogenous repair mechanisms with a sustained release of therapeutic agents (Bhatia, 2010; Wang L. et al., 2012). Although hundreds of biomaterials have been generated and investigated in the past decade, they can be generally categorized into the following categories, which will be discussed in relation to neurotrophin-based interventions below: microspheres, electrospun nanofibers and hydrogels (see Figure 2). The following studies are summarized in Tables 1, 2 to highlight past use of neurotrophins for regenerative interventions without (see Table 1) or with (see Table 2) biomaterial drug-delivery systems, respectively.

**TABLE 1:** Evidence supporting the neuroregenerative properties of classical neuroptrophin use in the context of treatments for CNS disease/injury.

**Neurotrophin**	**Species**	**Disease model/Injury**	**Intervention/Treatment**	**Neuroregenerative effect**	**References**
NGF	Rat	Unilateral lesion to nucleus basalis	Implantation of NGF-secreting fibroblasts	Enhanced NGF-induced axonal growth	Kawaja and Gage, 1991
	Rhesus Monkeys	Unilateral transection of the fornix	Implantation of NGF-secreting fibroblasts	Enhanced sprouting of cholinergic fibers	Kordower et al., 1994
	Rat	Complete crush injuries to the cervical DRG	Intrathecal NGF via osmotic minipumps	Regrowth of unmyelinated DRG fibers, restoration of behavioral sensitivity and stimulus-driven postsynaptic activity	Ramer et al., 2000
	Rat	Immunotoxic lesions to the CBF	Intracerebroventricular NGF via osmotic minipumps	Hypertrophy and enhanced axonal growth in CBF neurons, improved spatial orientation in a water maze	Winkler et al., 2000
BDNF	Rat	Optic nerve transection	Intravitreal BDNF injections	Enhanced local regrowth of RGC axons near the optic disc	Mansour-Robaey et al., 1994
	Mice	Striatal lesion	Intrastriatal infusion of BDNF antisense oligonucleotides via minipumps	Suppressed peri-wound dopaminergic sprouting	Batchelor et al., 2000
	Rat	PCA-induced lesions (forebrain or hippocampus)	BDNF infusions to lesion site via osmotic minipumps	Enhanced sprouting of 5-HT axons at the lesion site (both the forebrain and hippocampus)	Mamounas et al., 2000
	Rat	Complete transection of the thoracic spinal cord	Intrathecal BDNF via osmotic minipumps	Enhanced functional recovery of the hindlimb, modest sprouting of cholinergic fibers within injury cavity	Jakeman et al., 1998
	Rat	Photothrombotic stroke to the parietal cortex	Intravenous BDNF injections	Enhanced functional recovery of motor function, promoted widespread neuronal remodeling	Schäbitz et al., 2004
	Rat	Photothrombotic stroke to the parietal cortex	Intravenous BDNF injections	Enhanced functional recovery of sensorimotor function, neurogenesis (DG) and SVZ progenitor cell migration	Schäbitz et al., 2007
	Rat	MCAO via permanent intraluminal vascular occlusion	Intraventricular injections of CBD-bound BDNF	Enhanced SVZ progenitor cell proliferation, enhanced angiogenesis and improved neurological performance	Guan et al., 2012
	Rat	Endothelin-1-induced MCAO	Intraventricular infusion of BDNF antisense oligonucleotides via minipumps	Attenuated rehabilitation-mediated functional recovery of motor skills	Ploughman et al., 2009
	Mice	Photothrombotic stroke to the motor cortex	TrkB-Fc-embedded hydrogel into stroke cavity	Occluded AMPAkine-mediated and spontaneous functional recovery	Clarkson et al., 2011
NT-3	Mice	N/A	Conditional NT-3 knockout	Impaired NPC differentiation and LTP, deficits in spatial memory	Shimazu et al., 2006
	Rat	Partial bilateral transection of the thoracic spinal cord	Intrathecal NT-3 injection	Enhanced axonal sprouting of transected corticospinal tract	Schnell et al., 1994
	Rat	Partial bilateral crushing of the thoracic spinal cord	Intrathecal NT-3 via osmotic minipumps	Enhanced axonal growth within and outside the lesion site	Bradbury et al., 1999
		Focal hypoxic-ischemic brain injury	Implantation of NT-3-overexpressing NSCs	Promoted NSC differentiation into neurons, dampened glial scarring	Park et al., 2006
	Rat	Partial bilateral transection of the thoracic spinal cord	Implantation of NT-3-secreting fibroblasts	Enhanced axonal growth around and distal to lesion site, partial recovery of motor function	Grill et al., 1997

*DRG, dorsal root ganglion; CBF, cholinergic basal forebrain; RGC, retinal ganglion cells; PCA, *p*-chloroamphetamine; DG, dentate gyrus; SVZ, subventricular zone; MCAO, middle cerebral artery occlusion; CBD, collagen binding domain; TrkB-Fc, tropomyosin receptor kinase B-Fc; NPC, neural progenitor cell; LTP, long term potentiation; NSC, neural stem cells.*

**TABLE 2:** Examples of biomaterial-based neurotrophin treatments for regeneration and repair following CNS disease/injury *in vivo*.

**Neurotrophin**	**Species**	**Disease model/Injury**	**Biomaterial/Treatment**	**Effect of treatment**	**References**
NGF	Rat	Sciatic nerve transection	Nerve conduits loaded with NGF-embedded PLGA microspheres	Enhanced regeneration of motor and sensory axons	Santos et al., 2016
	Rat	Sciatic nerve transection	NGF-loaded PCL nerve conduit	Significantly enhanced mean axon diameter and myelin thickness, increased total number of regenerated fibers, enhanced recovery of electromyographic function	Liu et al., 2011
	Rat	Sciatic nerve transection	NGF-loaded chitosan conduit	Facilitated axonal regeneration, enhanced target muscle innervation and motor recovery	Wang H. et al., 2012
BDNF	Rat	Sciatic nerve transection	Nerve conduits loaded with NGF-embedded PLGA microspheres	Enhanced regeneration of sensory axons	Santos et al., 2016
	Mouse	Photothrombotic stroke to the motor cortex	Hyaluronan-based hydrogels loaded with BDNF	Improved recovery of motor function	Clarkson et al., 2015
	Mouse	Photothrombotic stroke to the motor cortex/L-NIO induced stroke	Hyaluronan-based hydrogels loaded with BDNF	Enhanced axonal sprouting, enhanced migration of neuroblasts to the peri-infarct cortex, enhanced recovery of forelimb function	Cook et al., 2017
	Rat	Acute brain injury via needle insertion	Electrospun PCL sacffolds embedded with a BDNF-mimetic	Redirected endogenous neuroblasts toward the scaffold, enhanced neuroblasts integration and promoted local neurite sprouting	Fon et al., 2014
NT-3	Rat	Complete transection of the thoracic spinal cord	Multichannel PLGA conduits loaded with rhNT-3	Enhanced axonal regrowth between spinal cord stumps, partial recovery of hindlimb locomotor function	Fan et al., 2011
	Rat	Partial bilateral transection of the dorsal spinal cord	Collagen-derived hydrogel loaded with NT-3	Enhanced regrowth of CST axons, mild improvements in motor function	Houweling et al., 1998
	Rat	Partial bilateral transection of the dorsal spinal cord	Acrylate-based hydrogel loaded with NT-3	Enhanced axonal outgrowth in the CST and raphespinal tract, recovery of locomotor function	Piantino et al., 2006

*rhNT-3, recombinant human neurotrophin-3; L-NIO, N5-(1-iminoethyl)-L-ornithine; CST, corticospinal tract.*

### Microsphere-Based Delivery Systems

Microspheres, also known as microparticles, are polymeric spheres with a diameter between one and 1000 μm. Historically, microsphere technology has been utilized for a diverse range of applications including cosmetics, electronics, and material engineering. In medical and scientific fields, microspheres are used to encapsulate drugs, and mediate their release to an injury site.

There are many techniques employed for microencapsulation of drugs including phase separation/precipitation, emulsion-solvent evaporation/extraction, spray drying and *in situ* polymerization (Jain, 2000). Each method has its own advantages and disadvantages, leaving the synthesis process to be chosen based on the application involved (i.e., polymer attributes, site of drug action, and therapy duration). The most common method of encapsulation is the emulsion-solvent extraction/evaporation method, which results in lyophilized ready to inject drug-loaded microspheres. In contrast to systemic methods of neurotrophin administration, microsphere-based drug delivery systems can be surgically injected straight into the injury site, avoiding issues with BBB passage. Microspheres also offer the advantage of being highly tunable with choice of polymer, particle size, and method of erosion all altering the rate of drug delivery from microspheres (Xu et al., 2002). Drug-loaded microspheres can also be injected into the peripheral circulation as they can cross the BBB. Furthermore, as loaded neurotrophins are encapsulated they are not enzymatically degraded until the microsphere degrades, thereby increasing the therapeutic half-life.

Santos et al. (2016) have illustrated the neurotrophic potential of PLGA microspheres loaded with NGF or BDNF. When applied to organotypic cultures derived from the SC and DRG, both of the loaded-microspheres enhanced neurite outgrowth when assessed 1 week after application. In contrast, free BDNF or NGF alone failed to show neurotrophic effects at this time, supporting the augmentation of neurotrophic effects as a direct result of microsphere encapsulation (Santos et al., 2016). In this same study, Santos et al. (2016) further confirmed a neurotrophic effect in a rat model of PNI. Briefly, neurotrophin encapsulated microspheres were loaded into a silicon tube filled with collagen gel before being injected into transected sciatic nerves. The use of retrograde tracers confirmed silicon tubes loaded with either NGF- or BDNF-loaded microspheres significantly increased the number of regenerated motor neuron axons in the SC, compared to control tubes loaded with free-floating neurotrophins. Furthermore, tubes loaded with encapsulated-NT-3 significantly increased the number of sensory axons in the DRG compared to free-floating controls (Santos et al., 2016). Similarly, sustained release of NGF from PLGA microspheres has also been reported by De Boer et al. (2010, 2011) with increased neurite outgrowth being observed as late as 35 days following subcutaneous implantation of the PLGA microspheres in rats. However, it should be noted that the long-term functional recovery observed in rats receiving the NGF-loaded microspheres was no greater than that of rats receiving autologous grafts (De Boer et al., 2011). The authors suggested that this may due to a “catch-up” or “blow through” effect, where intrinsic regeneration of the rat sciatic nerve is thought to be so effective that minor or early intervention-induced differences go unnoticed when long term regeneration is accessed (Young et al., 2001; De Boer et al., 2011). It is also possible that NGF exposure from the microspheres had stopped before a lasting NGF-mediated trophic effect could have been induced, resulting in a rebound effect.

The application of microsphere technology to stroke remains sparse due to several reasons. Whilst microspheres do support the sustained release of neurotrophins, the use of microspheres alone lack the mechanical support that benefits injuries with large lesion sites (i.e., stroke, SCI) (Sharp et al., 2012). Another major issue lies in the difficulty of sustaining the release of GFs in order to maintain levels within a given concentration range, an issue hampered by the challenge to create microspheres of a consistent diameter (Mohtaram et al., 2013). Further, it is known that there is a higher, initial burst of compound release from microspheres that restricts the overall duration and concentration of release (Binan et al., 2014). In attempts to overcome this, Bertram et al. (2010) have produced a PLGA-PLLA-PEG based microsphere delivery system that yields a consistent release of NGF without an initial burst, and with delivery lasting as long as 65 days. Nevertheless, the application of polymeric microspheres for therapeutic interventions has been found to be safe and well tolerated in human trials of brain tumors (Brem and Gabikian, 2001). Additionally, treatments utilizing neurotrophin-loaded microspheres in conjunction with other biomaterial scaffolds are emerging and showing promise (Xu et al., 2003; Lampe et al., 2011; Ju et al., 2014). For instance, Ju et al. (2014) investigated the therapeutic potential of loading PLGA microspheres with VEGF and Ang1, two factors that stimulate angiogenesis. The loaded microspheres were then embedded within a hyaluronic acid-derived hydrogel (containing anti-Nogo receptors) and injected into the stroke cavity of mice. Animals treated with the composite system showed enhanced angiogenesis around ischemic regions and facilitated recovery of locomotor function 6 weeks post-stroke. It is worth highlighting that to the best of our knowledge, no one has compared these hybrid delivery systems to systems containing the single biomaterial type, leaving the exact benefit of these hybrid models requiring future investigation.

### Electrospun Nanofibers and Conduits

Another fabrication technique that has proven useful in generating tunable, three-dimensional scaffolds consisting of micro-, and nano-sized fibers is electrospinning. Electrospinning was initially designed to generate textiles and filters before being proposed to produce wound dressing material (Doshi and Reneker, 1995). Nowadays, electrospun nanofibers are commonly used as neural scaffolds due to their ability to mimic the structure and surface-to-volume ratio of the ECM (Chew et al., 2005; Alhosseini et al., 2012; Jiang et al., 2012). Compared to other biomaterials, electrospun nanofibers offer the advantage of an easily adaptable shape and structure, reduced initial drug release and enhanced cell or drug attachment (Han et al., 2011). Conversely, electrospinning has proven difficult to maintain consistency in fiber sizes, with concerns also being placed on the use of toxic solvents involved in the synthesis of polymer-drug emulsions (Li et al., 2002).

Many groups have encapsulated neurotrophic factors within electrospun nanofibers and shown retention of neurotrophic factor bioactivity. For instance, Chew et al. (2005) showed the sustained release of NGF from *E-*caprolactone and ethyl ethylene phosphate (PCLEEP) electrospun copolymers. Three months after loading, NGF was still being released and capable of causing mild differentiative effects upon PC12 cells. PCL nanofibers have also been reported to release a NGF-BSA mixture for as long as 28 days, with significantly enhanced neurite outgrowth still being observed in PC12 cells (Valmikinathan et al., 2009). Similarly, electrospun PLC nanofibers that have been tethered to BDNF have been found to promote neurite extension and NSPC proliferation and differentiation into neurons and oligodendrocytes (Horne et al., 2009).

Electrospun scaffolds and conduits have been extensively studied as an intervention for SCI and peripheral nerve regeneration. In these injuries, axonal repair requires fine, ordered control to ensure functional recovery. Electrospun nanofiber scaffolds alone have already demonstrated the ability to promote stem cell proliferation and differentiation, and more importantly, enhance the outgrowth of new connections from neurons in the direction of aligned nanofibers (Yang et al., 2005; Corey et al., 2007; Xie et al., 2009). Fan et al. (2011) further illustrated the regenerative potential of a multichannel PLGA conduit loaded with recombinant human NT-3. Following complete transection of the thoracic SC in rats, use of retrograde tracing confirmed axonal regrowth between caudal and rostral SC stumps, with partial locomotor recovery also being observed 150 days post-injury. The use of NGF-loaded electrospun conduits has also been investigated in order to aid the regeneration of the PNS (Liu et al., 2011). Supporting this, chitosan-based conduits preloaded with NGF have been shown to facilitate the re-establishment of nerve function following injury to the sciatic nerves, with improved muscle innervation and functional recovery of motor function being observed 24 weeks after treatment (Wang H. et al., 2012). Furthermore, Liu et al. (2011) showed that PCL electrospun fibers containing NGF could promote nerve regeneration and improve electromyographic function in a rodent model of PNI. Amazingly, this electrospun treatment was found to provide the same degree of recovery as autografts, which is the current “gold standard” of nerve regeneration (Liu et al., 2011).

The use of neurotrophin-embedded electrospun fibers in the injured brain has been questioned due to the haphazard arrangement of the cerebrum and the invasive nature of electrospun conduit application. However, Fon et al. (2014) conducted a study to investigate the use of electrospun PCL scaffolds embedded with a small molecule BDNF-mimetic following acute injury to the brain. The PCL scaffolds were implanted toward the SVZ, with the needle tract resulting from implantation acting as the acute injury. Eight days after injury, authors found scaffold implantation with and without the BDNF mimetic, facilitated a significant increase in neuroblast migration from the rostral migratory stream (RMS) to the injury site. In contrast, at 21 days post-injury, a significant increase in neuroblast migration was only observed in animals treated with scaffolds containing the BDNF-mimetic (Fon et al., 2014). These findings suggest that electrospun fibers may be able to direct neurogenic processes to the injured brain, including influencing neuroblast infiltration into 3D biomaterial scaffolds. In further support of this notion, Rivet et al. (2015) investigated the use of a hybrid biomaterial system that utilized hydrogels loaded with PLLA-fibronectin electrospun fiber fragments. Sixty days after implantation into the striatum of rats, immunohistology revealed enhanced astrocyte and macrophage/microglia infiltration into the scaffolds. Whilst these findings support that resident cells of the brain were able to locate and utilize cues from the electrospun fibers, further works is needed to confirm the regenerative potential of these systems.

### Hydrogel-Based Neurotrophin Delivery Systems

First introduced as a novel material to construct soft contact lenses, Wichterle and Lim (1960) displayed the plausibility of using a porous material with low immunoreactivity in a living organism. Hydrogels have since become of immense interest as a vehicle for drug administration throughout the biomedical and pharmaceutical worlds. Hydrogels refer to three-dimensional, hydrophilic, polymeric networks that are capable of absorbing large amounts of water, or biological fluids (Peppas et al., 2000). Opposed to the fixed encapsulation of GFs, hydrogels allow neurotrophins to be blended into a polymer scaffold, resulting in a steady release of the neurotrophins from within the implantation site as the gel slowly biodegrades.

Compared to the other biomaterials, hydrogels offer many advantages for application in stroke treatments. Hydrogels typically express low toxicity, have a minimally invasive administration procedure, and have a relatively soft consistency that resembles neural tissue (Ratner and Hoffman, 1976). Additionally, *in situ* hydrogels show the unique ability to swell and fill the irregular conformations of stroke-induced lesions. This is hugely advantageous as it provides maximal mechanical and sustained pharmaceutical support directly to the damaged tissue. Specifically, drugs embedded into hydrogels have a sustained, consistent release into surrounding tissue lasting around 2–3 weeks (Tae et al., 2006; Overman et al., 2012; Clarkson et al., 2015; Cook et al., 2017). This overcomes various difficulties encountered by other delivery systems including increased adverse side effects resulting from higher doses and injuries acquired during repetitive drug administrations.

Hydrogels are known to yield anti-inflammatory and regenerative properties when used as a vehicle for NSPCs *in vivo*. For instance, a hyaluronan-heparin-collagen based hydrogel was able to promote survival of NSPCs and reduce infiltration of inflammatory cells following photothrombotic stroke in mice (Zhong et al., 2010). Similarly, Park et al. (2002) demonstrated the ability of a PGA-derived hydrogel to enhance neuronal differentiation and neurite elongation, reduce monocyte infiltration, and minimize glial scarring when used as a scaffold for NSPC application in a mouse model of hypoxic-induced ischemia. Further, the anti-inflammatory properties of hydrogels themselves have also been observed in rat models of SC injury (Woerly et al., 1998). These intrinsic properties of hydrogels make them ideal candidates as a drug-delivery system for neurotrophin-based treatments, in the hope to further maximize repair, and functional recovery.

The use of BDNF-embedded hydrogels has shown huge promise in repairing the ischemic brain and promoting functional recovery (Clarkson et al., 2015; Cook et al., 2017). A promising study from the Carmichael lab investigated the use of a BDNF-loaded hyaluronan-based hydrogel that was cross-linked with PEG, in two mouse models of stroke (Cook et al., 2017). In both models, treatment beginning at 1-week post-stroke was sufficient to induce significant axonal sprouting throughout cortical and cortico-striatal regions. These animals further displayed enhanced migration of neuroblasts to the peri-infarct cortex and improved functional recovery of forelimb activity. Worth noting, the regenerative potential of BDNF-loaded hydrogels has also been confirmed in aged mice receiving photothrombotic strokes, however, the magnitude of recovery was less than that of young mice (Clarkson et al., 2015). Hydrogels embedded with NT-3 have also displayed neuroregenerative properties when applied as a therapeutic intervention for SCI (Houweling et al., 1998; Piantino et al., 2006). Early work with a NT-3 embedded collagen-derived hydrogel revealed significant regrowth of CST axons following bilateral transection of the dorsal SC in rats (Houweling et al., 1998). In the same animal model, an acrylate-based hydrogel loaded with NT-3 was found to facilitate motor performance on the horizontal ladder walking task and the Basso-Beattie-Bresnahan locomotor score (Piantino et al., 2006). Collectively, the above highlights the benefits of hydrogel-derived delivery systems, providing sustained support to the injured brain and facilitating repair of nervous tissue.

### Heparan Sulfate Proteoglycans (HSPGs): A Promising Future Delivery System

The neuronal ECM is a complex and dynamic environment composed of a wide variety of glycoproteins and PGs. PGs are a group of proteins that consist of long, unbranched GAGs side chains covalently linked to a core protein. Interestingly, many GFs are known to require the presence of these extracellular GAGs in order to exert their biological function (Taipale and Keski-Oja, 1997; Whitelock and Iozzo, 2005).

**FIGURE 3:**
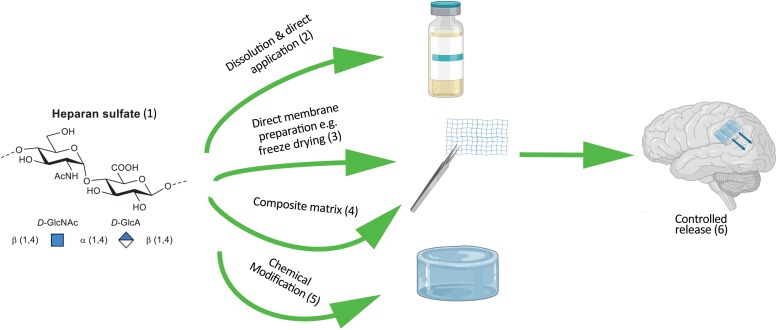
Schematic of incorporating HS into therapeutic devices. (1) Synthetic HS that approximates the degree of sulfation and acetylation of naturally occurring HS can be derived from heparin through chemical modifications. Example modifications of HS and composition of device. (2) HS adsorbed onto a collagen membrane. (3) Solid form of HS as a carbohydrate foam. (4) Electrostatically bound HS (net –ve charge) protein substrate (net +ve charge). (5) Pegylated, alkylated or alternative chemical modification of HS to control hydrophobicity and release for direct coating onto a device. (6) Device characteristics tuned to release HS into the extracellular matrix facilitating tissue regeneration.

Heparan sulfates are a subset of the class of linear GAG polysaccharide sugars. HS, as extracted from tissue sources and cleaved from its protein component, presents as a ∼15 kDa polymeric mixture. Constructed from a repeating disaccharide comprised of a glucosamine α – linked (1–4) to an uronic acid [glucuronic (*D-*GlcA) or the C-5 epimer iduronic (*L*-IdoA)]. Varying degrees and combinations of acetylation, sulfation or no-substitution at the amine moiety, as well as O-sulfation at the 2-position of the uronic acid and/or 6-position of the glucosamine gives rise to a considerable number of disaccharide permutations (Carnachan et al., 2016). These in turn can be combined into a vastly complex linear array. Enzymatic depolymerization generally reveals eight main disaccharide components (Carnachan et al., 2016), with the most abundant constituent being non-sulfated and comprising ∼30% of the population (as described in Figure 3).

Heparan sulfates are particularly evident during times of tissue growth and repair (Gallagher, 1989; Bernfield et al., 1999). Over the past decade the involvement of HS and/or HSPGs in cellular signaling mechanisms has become more and more evident. Many HSPGs are known to be involved in both synaptic and structural neuroplasticity, regulating synaptogenesis, LTP, and dendritic spine maturation (Ksiazek et al., 2007; Allen et al., 2012; Senkov, 2014). Several studies have also discovered HSPG-mediated potentiation of neurite outgrowth and cell migration *in vitro* (Walz et al., 1997; Hu, 2001; Jung Kim et al., 2003).

Many of the regenerative and reparative properties of HS are thought to be mediated through GAGs ability to act as a medium-affinity co-receptor for binding GF’s to their receptors (Bespalov et al., 2011; Chen et al., 2012). This GF-GAG interaction further enhances protein stability and bioavailability of GFs and their receptors (Chen et al., 2012, Chan et al., 2017). HSPGs are also highly sulfated giving the sugar a unique ability to create large binding pockets that can be utilized to house an abundance of GFs. Supporting this notion, early work from Kanato et al. (2009) used horizontal native- PAGE and gel chromatography analysis to confirm a direct molecular interaction between HS and the neurotrophins NT-3 and BDNF. The resulting complex was found to bind to both p75^NTR^ and TrkB, respectively, in a manner similar to polysialic acid, a protein that has been proposed to house and mediate BDNF concentrations and function (Kanato et al., 2008). Whilst this interaction remains to be identified both *in vitro* and *in vivo*, the above findings do support the idea that GAGs may regulate local GF concentrations, which could further serve to provide a reservoir of neurotrophins on cell surfaces or around the ECM. However, is should also be acknowledged that the exact role of HS with respect to its interaction with GFs, still requires future investigation (Lander, 2007).

The therapeutic use of HS remains sparsely investigated due to the low yield experienced when fractionating heparanoid raw materials (sourced as byproduct from Heparin manufacture). This is further complicated by limited supply and quality control of HS (Mulloy et al., 2016). The preparation of individual oligosaccharides remains highly costly, time consuming and difficult (DeAngelis et al., 2013), but recent advancements in technology have led to innovative synthetic or chemoenzymatic approaches allowing access to significantly longer purified HS structures (Kuberan et al., 2003; Liu et al., 2010; Tyler et al., 2015). However, the heterogeneity of native HS means synthesis of even a small portion of the structural diversity that is found in nature, even if limited in size to as small as an octasaccharide, is a daunting challenge.

Whilst literature investigating the ability of HS and heparin to cross the BBB is sparse, several studies suggest that these molecules may in fact cross the BBB. Early *in vitro* work has indicated Heparin of 3000 Da and smaller fragments (i.e., di, tetra, and hexasaccharides) can all cross a BBB barrier in a model system, without any obvious correlation with size to transport efficiency (Leveugle et al., 1998). Ma et al. (2002) have since reported BBB passage of small fragments of C3, a low molecular weight (2000 ± 200 Da) heparin-derived oligosaccharide designed for treatment of vascular dementia and Alzheimer’s disease. Specifically, C3 was able to penetrate the BBB and retain its’ inhibitory effect on factor Xa after systemic administration to non-human primates (Ma et al., 2002). Further supporting evidence has come from a recent study by Hippensteel et al. (2019) who found fluorescein-labeled HS fragments penetrated the BBB during sepsis and inhibited BDNF-mediated hippocampal memory. In addition, they further show that the binding affinity of HS fragments to BDNF increased with sulfation at the 2-O-position of iduronic acid and N-position of glucosamine (Hippensteel et al., 2019). These findings support that sulfation at these sites is critical for memory impairments, although it has not been shown if this is the case for all cognitive domains. Whilst, the involvement of these sulfation sites in post-stroke motor and sensory impairments is yet to be investigated. Given that poor BBB passage is thought to be an underlying cause for a lack of clinical translation of compounds targeting the CNS (Pardridge, 2005), HS may prove extremely useful as a backbone for therapeutic interventions targeting the injured brain.

Although HS application into CNS disease is extremely limited so far, a recent study by Hill et al. (2012) illustrated the neuroregenerative potential of the HSPG glypican, in the MCAo model of stroke. Intracerebral glypican was administered through micro-osmotic pumps for a week following stroke, which resulted in a significant decrease in GFAP and increased MAP2 expression in the peri-infarct region. These findings support a glypican-mediated reduction in astrocyte activation and enhanced neuronal survival, respectively. Furthermore, when put through various sensorimotor tasks glypican-treated animals performed significantly better when assessed 10- and 14-days after stroke, compared to control animals (Hill et al., 2012). Another HSPG, Perlecan domain V, has also been shown to afford neuroprotection in both young and aged mice (Bix et al., 2013). Perlecan domain V has also been shown to regulate both VEGF and BDNF levels (Lee et al., 2011; Bix et al., 2013). Whilst the regenerative potential of Perlecan domain V when administrated via a hydrogel or other biomaterial has yet to be studied, it is likely that similar if not better effects would be observed compared to when Perlecan domain V is given systemically.

A key component of biomaterial design lies in the topography and three-dimensional structure of its’ scaffold (Führmann et al., 2017). Given the physiological role of HS chains in supporting the ECM of the CNS, the incorporation of HS chains into biomaterial scaffolds has been of interest in the field of regeneration and repair. Fibrin glue loaded with a HS scaffold has been shown to increase mineralized bone deposition and osteogenic marker expression, reflecting enhanced bone repair (Woodruff et al., 2007). Microcomputed tomography further revealed almost complete closure of chronic cranial defects sites in rats 3 months after treatment with the fibrin-HS glue, compared to minimal healing seen in fibrin glue without HS chains (Woodruff et al., 2007).

Heparan sulfates, as the sodium salt, demonstrates very good water solubility and only when solutions get above 10% w/w does any increase in viscosity become apparent. In an anhydrous form it will reach an equilibrium water-content of 13% at room temperature, which must be accommodated for (or freshly freeze-dried material must be used) or else dose control in constructs becomes erroneous (Murali et al., 2013). Other salt-forms are generally more problematic in terms of physical handling; the pyridinium salt, for example, being very hygroscopic. This solubility means a preformed porous substrate can be dosed with a solution (Figure 3, direct application) prior to wound inclusion (Murali et al., 2013). Alternatively, solid support foams can readily be prepared through freeze drying a solution of neat HS, or by combining HS with other materials in solution to form a stable solid matrix (Figure 3, composite matrix and or membrane preparation). Pre-formed microspheres or membranes generated through electrospinning as depicted in Figure 2 may have the HS included during preparation and/or adsorbed into the construct or coated onto the material surface.

Collagenous matrices with covalently attached HS chains have also been found to promote matrix integrity, facilitate angiogenesis and reduce foreign body reactions when implanted subcutaneously into rats (Pieper et al., 2000). Early *in vitro* work has also confirmed a trophic effect of HS-incorporation on cells of the CNS. Guan et al. (2013) discovered that a chitosan-gelatin scaffold containing GAGs, HA, and HS, promoted NSC/NPC adhesion, survival and long-term growth. A similar finding was observed by Uygun et al. (2009) who recorded increased MSC density, migration, and proliferation of MSCs cultured on chitosan membranes modified with HS chains. The trophic potential of HS-embedded biomaterial scaffolds has also been acknowledged by Mammadov et al. (2012), who illustrated that combining a heparan-sulfate-mimicking peptide nanofibers with a laminin-derived nanofiber scaffold significantly enhances neurite outgrowth of PC-12 neurons. The above literature supports the use of HS-seeded biomaterials for neurotrophin-mediated regeneration, leaving the translational potential of such treatments to future research. It is also worth noting that in the body, HS chains are manufactured as a PG, which are present in the ECM at relatively high concentrations compared to free HS fragments (i.e., mg/mL. PPM) (Lander and Selleck, 2000). Certainly, it is recognized that the shedding of HS chains from HSPGs can release sequestered GFs to stimulate GF activity (Yu et al., 2017). However, it remains a matter of debate as to whether or not all GF-GAG interactions are mediated by HS chains or the full HSPG (i.e., HS chains and protein core). For example, the HSPG, glypican, requires the presence of its HS chains in order to facilitate FGF-2 (Song et al., 1997; Lander and Selleck, 2000) but not BMP signaling (Kirkpatrick et al., 2006). Furthermore, the protein core of glypican has been found to bind and promote Hedgehog (Hh) signaling without the involvement of HS chains (Capurro et al., 2008). Future work elucidating the exact molecular mechanism associated with this GF-HS interaction will help guide the development of HS-embedded biomaterials for therapeutic application.

## Concluding Remarks

Neurodegenerative diseases, such as stroke, remain the leading cause of adult death and disability worldwide. With the aging population on the rise, and these diseases preferentially affecting the elderly, there is a growing interest in the development of interventions that can fight against neurological decline and enhance functional recovery. Protective therapies for stroke are massively limited due to the narrow window of opportunity that is required for success. Consequently, the generation of regenerative interventions have become of utmost importance to help treat stroke at a clinically relevant time point, and to successfully translate findings from bench to bedside.

After decades of preclinical studies and still no successful treatments for stroke recovery in humans, researchers have agreed that future work investigating the neurobiology, and progression of post-stroke recovery (both spontaneous and treatment-induced) will be crucial on the journey toward the identification of key molecular targets and an optimal time of intervention (Bernhardt et al., 2017; Corbett et al., 2017). Armed with this knowledge, the scientific and medical worlds will have the greatest chance of providing maximal recovery from stroke in multidisciplinary phase II clinical trials.

Neurotrophins have shown great promise in modulating the injured brain and promoting functional recovery in preclinical models of neurodegenerative disease. However, the clinical success of these treatments is yet to be observed due to poor BBB passage, short half-life and lack of sufficient quantities for clinical use. The use of biomaterials creates a unique delivery system that alters the pharmacokinetic profile of neurotrophins and offers a mechanism to penetrate the BBB and treat the injured brain with concentrations that are only a fraction of those used during systemic administration. Recent animal-based experimental research has shown great promise using biomaterial-delivery systems to deliver neurotrophins to enhance functional recovery, with each biomaterial type yielding distinct properties that suit different biomedical applications. For example, the linear nature of electrospun conduits has shown the most success in promoting controlled nerve regeneration in animal models of SCI (Fan et al., 2011; Wang H. et al., 2012). Alternatively, the ability of hydrogels to expand and fill irregular injury sites in conditions such as stroke has proven extremely useful for providing additional mechanical stability that other biomaterial systems lack.

Heparan sulfates offers a new therapeutic potential in promoting rapid tissue regeneration. It has shown significant benefit in repair of large defects in controlled wound sites (Murali et al., 2013; Wang et al., 2014) yet its full therapeutic benefit is unexplored, in no small way due to availability and reliability of supply (Mulloy et al., 2016). The versatility of this biopolymer, intrinsic to cellular regulation, is well suited to biomaterial applications *in vivo*: it is extremely well tolerated, readily decomposed by native enzymatic processes, is compatible with accepted medical device materials (e.g., collagen sponge or hydrogels) and can be included into device technologies through a variety of mechanisms.

Of note, an exciting biomaterial approach that is emerging involves not just one, but many biomaterial types combined into a hybrid delivery system. Early work with this technology has shown the fine-tuned release of multiple GFs over different release profiles (Burdick et al., 2006). Specifically, the sequential release of glial cell-derived neurotrophic factor (GDNF)- and BDNF-loaded PLGA microspheres was achieved by loading neurotrophins to opposite ends of a PEG-based hydrogel implant (Lampe et al., 2011). Healthy rats given the combinational treatment, with the implant being applied to target the striatum (BDNF) and sustantia nigra (GDNF) with the hope of assisting transplant survival and long-term axon extension, respectively, in future work with animal models of Parkinson’s disease. Results showed that GDNF was released within 28 days of implantation, whereas BDNF release was observed as late as 56 days. Due to the recency of the development of these sequential drug-delivery systems, they have sparsely been investigated in animal models of CNS injury or disease. Nonetheless, given the complex spatiotemporal, pathological events that occur in the days to weeks to months following neurodegenerative diseases, these delivery systems will yield great promise with delivery of multiple GFs to appropriate regions of the brain at relevant therapeutic time points to maximize recovery.

To ensure such treatment options translate into the clinic, much needed follow-up studies are required and clinical validation studies to be undertaken. Given the knowledge we have already gained around neurotrophic signaling especially with respect to BDNF-mediated improvements in functional recovery and the advances made in the biomaterial-delivery field, there is an exciting prospect of being able to deliver a potential treatment to contribute toward recovery from stroke and other neurological disorders.

## Author Contributions

JH, NA, SH, and AC wrote and edited the final version of the manuscript. JH prepared the figures. AC conceived the idea for the review of the manuscript.

## Conflict of Interest Statement

The authors declare that the research was conducted in the absence of any commercial or financial relationships that could be construed as a potential conflict of interest.

## Abbreviations

AAV: adeno-associated viral vector
ALS: amyotrophic lateral sclerosis
AMPA: α-amino -3-hydroxy-5-methyl-4-isoxazolepropionic acid
Ang1: angiopoietin-1
BBB: blood-brain-barrier
BDNF: brain-derived neurotrophic factor
BMA: basilar membrane area
BMP: bone morphogenic protein
BSA: bovine serum albumin
CMT1A: Charcot-Marie-Tooth type 1A
CNS: central nervous system
CREB: cAMP-response element-binding protein
CST: corticospinal tract
DAG: diacylglycerol
DRG: dorsal root ganglion
ECM: extracellular matrix
ERK: extracellular signal-regulated kinase
FGF: fibroblast growth factor
GABA: gamma-Aminobutyric acid
GAG: glycosaminoglycan
GAP43: growth associated protein 43
GFs: growth factors
GFAP: glial fibrillary acidic protein
GSK-3: glycogen synthase kinase 3
HS: heparan sulfate
HSPG: HS containing proteoglycan
IP3: inositol triphosphate
JNK: c-JunN-terminal kinase
LTP: long-term potentiation
MAP-2: microtubule-associated protein-2
MAPK: mitogen-activated protein kinase
MSC: mesenchymal stem cells
MCAo: middle cerebral artery occlusion
NADE: p75^NTR^-associated cell death executor
NF-kB: nuclear factor kappa-light-chain-enhancer of activated B cells
NGF: nerve growth factor
NIS: Neuropathy Impairment Score
NPC: neural progenitor cell
NRIF: neurotrophin-interacting factor
NT-3: neurotrophin-3
NSC: neural stem cells
NSPC: neural stem progenitor cell
p75NTR: p75 neurotrophin receptor
PI3K: phosphatidylinositol-3 kinase
PAGE: polyacrylamide gel electrophoresis
PCL: poly(ε-caprolactone)
PEG: polyethylene glycol
PG: proteoglycan
PGA: poly y-glutamic acid
PKC: protein kinase C
PLA: poly(lactic acid)
PLLA: poly-L-lactide
PLC- γ 1: phospholipase C- γ 1
PLGA: poly(lactide-co-glycolide)
PNI: peripheral nerve injury
PNS: peripheral nervous system
PPE: poly(phosphoester)
RhoA: ras homolog gene family member A
SC: spinal cord
SC-1: Schwann-Cell 1
SCI: spinal cord injury
SVZ: subventricular zone
SGZ: sub granular zone
SNP: single nucleotide polymorphism
TBI: traumatic brain injury
TFs: transcription factors
Trk: tropomyosin receptor kinase
VEGF: vascular endothelial growth factor.
